# Strategies to improve the efficiency of mesenchymal stem cell transplantation for reversal of liver fibrosis

**DOI:** 10.1111/jcmm.14115

**Published:** 2019-01-11

**Authors:** Chenxia Hu, Lingfei Zhao, Jinfeng Duan, Lanjuan Li

**Affiliations:** ^1^ Collaborative Innovation Center for Diagnosis and Treatment of Infectious Diseases, State Key Laboratory for Diagnosis and Treatment of Infectious Diseases, School of Medicine, First Affiliated Hospital Zhejiang University Hangzhou Zhejiang PR China; ^2^ Kidney Disease Center, First Affiliated Hospital, College of Medicine Zhejiang University Hangzhou Zhejiang PR China; ^3^ Key Laboratory of Kidney Disease Prevention and Control Technology Hangzhou Zhejiang PR China; ^4^ Institute of Nephrology Zhejiang University Hangzhou Zhejiang PR China; ^5^ The Key Laboratory of Mental Disorder Management of Zhejiang Province, Department of Psychiatry, First Affiliated Hospital Zhejiang University Hangzhou Zhejiang PR China

**Keywords:** improvement, liver fibrosis, mechanism, mesenchymal stem cell, regression

## Abstract

End‐stage liver fibrosis frequently progresses to portal vein thrombosis, formation of oesophageal varices, hepatic encephalopathy, ascites, hepatocellular carcinoma and liver failure. Mesenchymal stem cells (MSCs), when transplanted in vivo, migrate into fibrogenic livers and then differentiate into hepatocyte‐like cells or fuse with hepatocytes to protect liver function. Moreover, they can produce various growth factors and cytokines with anti‐inflammatory effects to reverse the fibrotic state of the liver. In addition, only a small number of MSCs migrate to the injured tissue after cell transplantation; consequently, multiple studies have investigated effective strategies to improve the survival rate and activity of MSCs for the treatment of liver fibrosis. In this review, we intend to arrange and analyse the current evidence related to MSC transplantation in liver fibrosis, to summarize the detailed mechanisms of MSC transplantation for the reversal of liver fibrosis and to discuss new strategies for this treatment. Finally, and most importantly, we will identify the current problems with MSC‐based therapies to repair liver fibrosis that must be addressed in order to develop safer and more effective routes for MSC transplantation. In this way, it will soon be possible to significantly improve the therapeutic effects of MSC transplantation for liver regeneration, as well as enhance the quality of life and prolong the survival time of patients with liver fibrosis.

## 1 INTRODUCTION

The liver is a digestive organ that stores glycogen, scavenges toxins and participates in protein synthesis for metabolic homeostasis. Because it makes direct contact with external toxins, the liver is easily injured under stress conditions. In response to repeated and chronic liver injury induced by hepatitis B virus (HBV), hepatitis C virus (HCV), sustained alcohol consumption and fat deposition,^1^, ^2^, ^3^, ^4^ the liver can accumulate aberrant myofibroblasts and extracellular matrix, thus generating liver fibrosis with poor prognosis. The pathogeneses of primary biliary cirrhosis, primary sclerosing cholangitis and autoimmune hepatitis are very different from other types of chronic liver fibrosis, as portal fibroblasts are found around bile ducts.^5^ Intriguingly, schistosomiasis induces liver fibrosis through accumulation of parasitic ova and periovular granulomas in portal veins.^6^ Hepatolenticular degeneration, known as Wilson’s disease, accounts for a small proportion of metabolic liver diseases. It is caused by a mutation in the Wilson disease protein (*ATP7B)* gene and frequently leads to liver fibrosis.^7^ In addition, metabolic syndromes including obesity, insulin resistance and diabetes have recently been found to be closely related to end‐stage liver fibrosis.^8^ Although the mortality of liver cirrhosis varies substantially across different regions of the world, it has been universally acknowledged by multiple investigators that liver cirrhosis has gradually become an increasing health burden worldwide, as liver cirrhosis and other chronic liver diseases contributed to 2% of deaths worldwide in 2015, with a relative increase of 10.3% from 2005.^9^ They also highlighted that the increasing mortality mainly attributed to viral hepatitis, alcoholic liver disease and non‐alcoholic fatty liver disease in developed countries.^9^ The long‐term inflammatory response and fibrotic state induced by various factors leads to other complications, including hepatocellular carcinoma (HCC) and liver failure.

Although multiple drugs are available for recovering liver function in patients, there are almost no effective drugs for reversing the pre‐existing accumulation of myofibroblasts and extracellular matrix. Currently, the most effective treatment for end‐stage liver fibrosis is liver transplantation, but it is limited by scarce donor grafts, immunologic rejection, complex surgery, high costs, etc. Although hepatocyte transplantation, which emerged as a substitution, is able to restore liver function and promote liver regeneration, this treatment is limited because hepatocytes easily lose their viability in vitro. Transplantation of stem cells, including mesenchymal stem cells (MSCs), haematopoietic stem cells and endothelial progenitor cells, has proven to be effective in eliminating chronic liver injury to repair fibrotic livers by promoting hepatocyte transdifferentiation and hepatocyte proliferation, inhibiting activated hepatic stellate cells (HSCs), up‐regulating the activity of matrix metalloproteinases (MMPs) and promoting neovascularization in liver tissues.^10^ However, it is hard to regress more significant liver fibrosis (cirrhosis), thus an intervention that targets the fibrosis is needed. Considering that MSCs have abundant resources, strong proliferative ability, multilineage potential and no ethical considerations for widespread application to repair various organ injuries, they are currently transplanted in vivo to reduce hepatocyte apoptosis and promote hepatocyte regeneration.^11^ Before application, the isolated and purified MSCs must met three criteria according to the International Society for Cellular Therapy: adherence to plastic under standard culture conditions; expression of CD105, CD73 and CD90, and lack of expression of haematopoietic and endothelial markers including CD11b, CD14, CD31, CD34, CD45 and HLA‐DR; differentiation into adipocytes, osteocytes and chondrocytes under specific in vitro culture conditions.^12^ However, only a small number of MSCs migrate to injured tissues after cell transplantation, so multiple studies have tried to investigate effective strategies for improving the survival rate and activity of MSCs to treat liver fibrosis. Repairment of the injured tissues of liver fibrosis is influenced by multiple factors including the delivery route, the resources of transplanted cells, the number of infused cells, culture conditions, gene modification of MSCs and other potential factors. Hence, we herein arrange and analyse the current evidence related to MSC transplantation in liver fibrosis and summarize the detailed mechanisms and new strategies of MSC transplantation for promoting the regression of liver fibrosis. We anticipate the development of safer strategies to improve MSC activities in vivo to repair liver function and promote the regression of liver fibrosis in regenerative medicine.

## 2 POTENTIAL MECHANISMS

**Figure 1:**
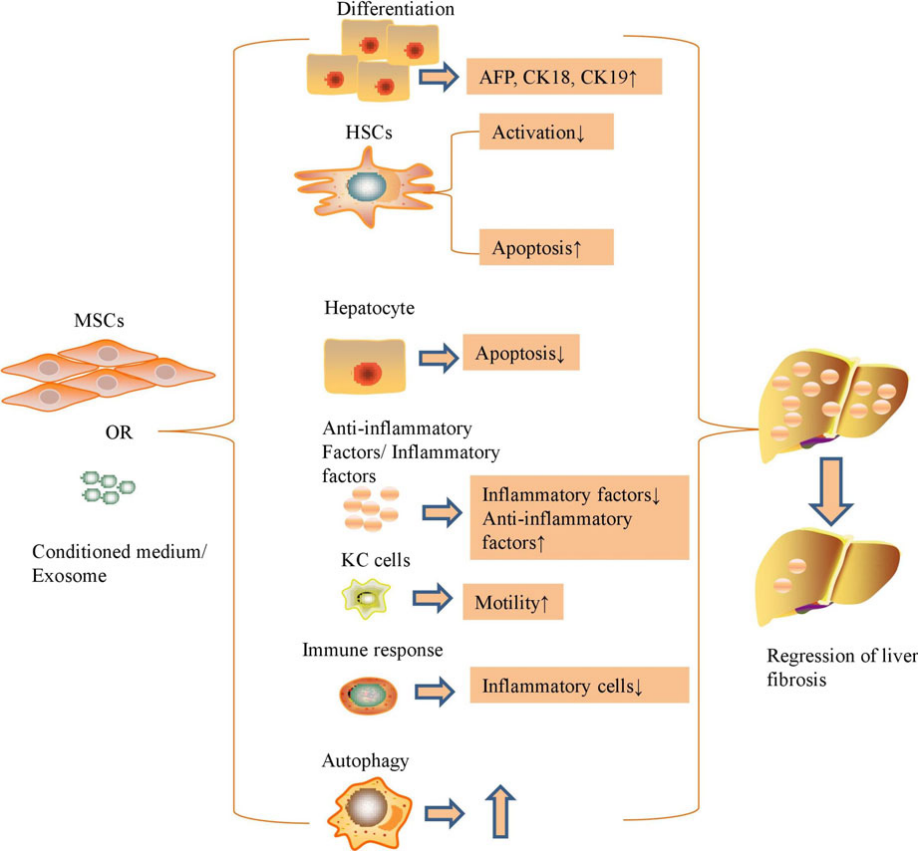
The potential mechanisms of MSC and MSC derivative‐based therapies in liver fibrosis for enhancing the therapeutic effects

Chronic liver injury induces liver fibrosis via up‐regulating the accumulation of extracellular matrix in vivo, and then normal hepatic architecture is replaced by a nodular structure of fibrous septa. In general, myofibroblasts are the major source of extracellular matrix, HSCs are considered to be the principal precursor population for myofibroblasts. Because liver tissue consists of multiple cell types, in vitro studies do not completely mimic the complex situation of the liver, but animal models can be used as a gold standard for in vivo study. A majority of studies investigated the potential mechanisms of MSC and MSC derivative‐based therapies in liver fibrosis for enhancing the therapeutic effects (Figure 1).

Mesenchymal stem cells migrate into fibrogenic liver tissues after transplantation in vivo and then differentiate into hepatocyte‐like cells or fuse with hepatocytes to protect liver function.^13^ Interestingly, Park et al previously showed that human palatine tonsil‐derived MSCs responded only to disease tissue, as they were detected in damaged livers but not in healthy livers, and it was also shown that these implanted MSCs differentiated into hepatocyte‐like cells to eliminate liver fibrosis via activation of autophagy and down‐regulation of the TGF‐β signalling pathway.^14^ In contrast, menstrual blood‐derived stem cells were demonstrated to eliminate collagen deposition and inhibit proliferative HSCs via paracrine mediators, but few of the transplanted cells differentiated into functional hepatocyte‐like cells despite migrating to the sites of injury.^15^ Transplanted MSCs up‐regulate the expression levels of key enzymes associated with glucose metabolism in carbon tetrachloride (CCl_4_)‐induced liver cirrhotic rats, thus maintaining normal metabolic status in liver fibrotic rats.^16^ Transplantation of MSCs consequently improved liver function and reduced liver histopathology and hepatobiliary fibrosis by inhibition of HSCs, down‐regulation of collagen deposition and enhancement of extracellular matrix remodelling via the up‐regulation of MMP‐13 and down‐regulation of tissue inhibitor of metalloproteinase (TIMP)‐1.^17^ Coculture of MSCs and HSCs inhibited the proliferation of HSCs and promoted cell apoptosis of HSCs via down‐regulating the E3 ligase S‐phase kinase‐associated protein 2 (SKP2) level, attenuating the ubiquitination of p27 and increasing the stability of p27.^18^ Moreover, MSCs are demonstrated to produce various growth factors and cytokines with anti‐inflammatory effects in vitro and in vivo to reverse the liver fibrotic state, as transplantation of MSCs increases the serum levels of vascular endothelial growth factor (VEGF), hepatocyte growth factor (HGF), IL‐10 and MMP‐9 in injured livers.^19^ MSCs in vivo attenuated hepatic fibrosis as shown by decreased serum levels of collagen I, type III procollagen, collagen IV, hyaluronic acid and laminin, and down‐regulated liver collagen proportionate area, hepatic hydroxyproline and liver α‐smooth muscle actin (α‐SMA); this progress is accompanied by reduced expression of serum TGF‐β1 and reduced hepatic levels of TGF‐β1, Smad3 and Smad4 but increased Smad7 expression.^20^, ^21^ MSCs significantly ameliorated liver fibrosis in mice via stimulation of interferon (IFN)‐γ and inhibition of lymphocyte proliferation,^22^ and MSCs has been proved to enhance the release of serum interleukin (IL)‐4 and IL‐10 and improve the motility of Kupffer cells for elimination of liver fibrosis in rats.^23^ MSCs significantly decreased the serum level of inflammatory IL‐17 and the number of IL‐17 producing Th17 cells, while increased serum levels of immunosuppressive IL‐10, indoleamine 2,3‐dioxygenase (IDO), kynurenine and number of CD4^+^ IL‐10^+^ T cells for attenuating liver fibrosis.^24^ MSCs transplanted in vivo were also shown to significantly suppress the activity of microRNA‐199 and increase the level of liver keratinocyte growth factor,^25^ which then increases the level of liver microRNA‐125b to suppress the activation of the Hedgehog signalling pathway in the injured liver, thus ameliorating experimental cirrhosis.^26^ Adipose‐derived MSCs are able to reverse pathological changes in non‐alcoholic steatohepatitis‐induced cirrhosis by reducing the number of inflammatory cells, including intrahepatic infiltrating cells, and reducing the ratio of CD8^+^/CD4^+^ cells.^27^

MSCs directly or indirectly eliminate the deposition of extracellular matrix by hepatogenic differentiation, fusion with hepatocytes, paracrine effects and immunological regulation, contribute to degradation of scar tissue and promotion of myofibroblast apoptosis. All of these potential mechanisms cooperate to regulate the therapeutic effects of MSC transplantation in liver cirrhosis models.

## 3 ROUTES OF TRANSPLANTATION

As MSC transplantation can be applied via different routes and acquire different efficiency, the optimal way to transplant MSCs may significantly improve the prognosis of liver fibrosis. However, the optimal route for MSC transplantation in the regression of liver fibrosis remains controversial. Truong et al showed that transplantation of MSCs by not only the tail vein but also the portal veins effectively improved liver function and protected the liver from continued development of fibrogenesis.^28^ Intravenous and intrasplenic MSC transplantation demonstrated a comparable restoration of liver function, but the intravenous route significantly decreased the levels of IL‐1β, IL‐6 and INF‐γ in liver tissues than the intrasplenic route.^29^ Wang et al demonstrated that intraportal transplantation was a better route than tail vein transplantation for improving the therapeutic effects of MSCs on liver fibrosis.^30^ MSC transplantation via portal vein significantly decreased hepatic arterial perfusion index, but increased portal vein perfusion and total liver perfusion as shown by computed tomography perfusion scan; moreover, liver functional test and histological findings in portal vein group were significantly improved but there seemed to be no significance in rats receiving MSCs from tail vein.^30^ Zhong et al detected no signal change in the vena caudalis group by magnetic resonance imaging, but they showed that MSC migration was gradually increased after immediate transplantation and decreased gradually after 3‐hour transplantation through portal vein route. Then, MSC signals disappeared in tissues around portal area while appeared in fibrous tuberculum at the edge of the liver at day 14 after transplantation. They concluded that portal vein route seemed to be more beneficial than the vena caudalis route on MSC migration to fibrotic liver.^31^ Comparing intravenous, intrahepatic and intraperitoneal injection routes, Zhao et al demonstrated that intravenous injection was the most effective route for improving serum IL‐10 levels and decreasing IL‐1β, IL‐6, tumour necrosis factor (TNF)‐α and TNF‐β in liver tissue to reverse liver fibrosis and restore liver function.^32^ In addition to the transplantation route, the transplantation frequency can be changed for improving the transplantation efficiency. Repeated infusions of MSCs three times significantly improved survival, liver fibrosis and necrosis than infusions of the same number of MSCs in a single dose. The repairment was accompanied by up‐regulation of the fibrogenic‐related genes and improved homing of MSCs after long‐term observation for 3 weeks.^33^ According to current studies, the majority of investigators accept that portal vein route seems to be the best choice for MSC transplantation. However, the optimal route for transplantation in animal models still needs to be further investigated to achieve better effects in clinical trials.

## 4 SOURCES OF MSCS

Considering that MSCs from different sources may have unique features, specialists have compared the therapeutic effects of different MSCs at the molecular level and pathological process. Sayyed et al demonstrated that umbilical cord blood‐derived CD34^+ ^MSCs were more efficient than bone marrow‐derived MSCs in elevating albumin level and reducing alanine aminotransferase (ALT) level, and they concurrently showed that umbilical cord blood‐derived CD34^+^ MSCs reduced the levels of COL1A1, TGF‐β1 and α‐SMA to a lower degree and increased the level of MMP‐9 to a greater degree than bone marrow‐derived MSCs.^34^ Rengasamy et al suggested that bone marrow‐derived MSCs were more effective in reducing liver fibrosis than Wharton’s jelly‐derived MSCs in CCl_4_‐induced liver fibrotic rats, as shown by lower levels of α‐SMA, higher levels of MMP‐1 and greater activation of hepatic progenitor cells in rats treated with bone marrow‐derived MSCs.^35^ Hao et al found that adipose‐derived MSCs achieved greater reductions of the proliferation and activation of HSCs and secreted higher levels of nerve growth factor and TGF‐β1 in the cell culture medium than bone marrow‐derived MSCs. They also found that although adipose‐derived MSCs improved anti‐inflammatory and anti‐fibrotic effects than bone marrow‐derived MSCs, the differences in inflammatory activity and fibrosis staging scores were not significant. Thus, they concluded that bone marrow‐derived MSCs and adipose‐derived MSCs are similarly effective at attenuating liver fibrosis.^36^ In contrast, Baligar et al demonstrated that bone marrow‐derived CD45^+ ^MSCs had better anti‐fibrotic ability than adipose‐derived MSCs because they expressed higher levels of MMP‐9 and MMP‐13 and inhibited HSC proliferation more effectively.^37^ Most comparisons were conducted between bone marrow‐derived MSCs and other MSCs, while the optimal choice is still undetermined for treating liver fibrosis. To this end, we want to highlight that bone marrow‐derived MSCs are applied as the main source of MSC transplantation, hence, we call for more studies to compare the advantages and disadvantages among MSCs from various resources.

## 5 MSC‐DERIVED SOURCES

Transplantation of hepatogenic MSCs, which are induced towards hepatocyte‐like cells in the presence of several hepatogenic factors,^38^ also exerts antifibrotic effects on liver cirrhosis. Transplantation of hepatogenic MSCs up‐regulated the levels of HGF, Bcl‐2, hepatocyte nuclear factor‐4α, FOXa2 and CYP7a1 in liver tissue, while decreasing the levels of serum fibronectin and hepatic TNF‐α, TGF‐β1, β‐5‐Tub and α‐fetoprotein (AFP) levels in animal models of liver fibrosis.^39^ Awan et al transdifferentiated MSCs into hepatic oval‐like cells in vitro and transplanted these cells into fibrotic livers, showing that these cells had higher homing rates and stronger effects on recovery from liver fibrosis than undifferentiated MSCs.^40^ However, there is still controversy surrounding the beneficial effects of these two kinds of cell transplantation. MSCs are more effective in maintaining liver function compared with hepatogenic MSCs, although both of them can effectively reverse liver fibrosis in a rat model.^41^

The use of secretome derived from MSCs or conditioned media to reduce liver fibrosis has gradually become a hot topic in current regenerative medicine. The secretome is a special set of factors (soluble proteins, free nucleic acids, lipids and extracellular vesicles) secreted into the extracellular space and changes in response to fluctuations in various conditions.^42^ Cell‐free secretome isolated from MSCs exerted antifibrotic effects by inhibiting activation of TGF‐β/Smad signalling and HSCs.^43^ Exosomes, which are small (30‐100 nm in diameter) membrane vesicles released by MSCs from various resources, are proved to have the same functions in vivo with their derived cells.^44^ They are also able to reduce surface fibrous capsules, soften textures and alleviate inflammation and collagen deposition in fibrotic livers by inhibiting epithelial‐to‐mesenchymal transition.^45^ Conditioned medium from MSCs induces apoptosis of HSCs, protects hepatocytes from apoptosis and down‐regulates the number of infiltrating macrophages, thus exerting antifibrotic effects and healing fibrotic scarring in liver tissue.^46^, ^47^ Conditioned medium derived from MSCs was demonstrated to contain high levels of anti‐apoptotic factors such as IL‐6 and insulin growth factor binding protein 2 and anti‐inflammatory factors such as interleukin‐1 receptor antagonist (IL‐1Ra).^48^ Conditioned medium of MSCs attenuated liver fibrosis by reducing IL‐17 and number of Th17 cells, but the protective effects of MSCs and conditioned medium of MSCs can be completely abrogated by IDO inhibitor.^24^ Moreover, Zhang et al used three‐dimensional (3D) culture condition to proliferate MSCs in vitro, and they found that conditioned medium of 3D‐cultured MSCs protected hepatocytes from CCl_4_‐induced injury and apoptosis more effectively than conditioned medium from general cultured MSCs.^49^ Furthermore, Liang et al fabricated a nanoparticle that carries the regenerative factors derived from MSCs and further coated it with the membranes of red blood cells to increase blood stability. Then they demonstrated that transplantation of these analogues significantly preserved the normal hepatic lobule structures and enhanced liver regeneration in the mouse with CCl4‐induced liver injury when compared to transplantation with conditioned medium or nanoparticles.^50^ The therapeutic potency of MSC‐based cell‐free therapy presents exciting new avenues for intervention in liver fibrosis largely via the constant transfer of miRNAs and proteins to regulate different pathways.

## 6 MODIFICATION OF THE MSC MICROENVIRONMENT

Transplanted MSCs encounter a toxic and inflammatory microenvironment that causes many active MSCs to undergo cell death, thereafter, only a small number of MSCs migrate into injury sites after cell transplantation. The microenvironment around MSCs always influence MSC activities and promote the proliferation or differentiation of MSCs, thus various treatment in vitro and in vivo emerged as good strategies to improve MSC transplantation efficacy (Table 1).

**Table 1:** Modification of the microenvironment to improve MSC transplantation efficacy in liver fibrotic models

Dose	Modification	Timing of treatment	Receptor	Route	Cause	Animal	MSC source	Effect	Mechanisms	Ref
1 × 10^6^	3D culture	Pretreatment	MSCs	Tail vein	CCl_4_	Mice	Adipose	Collagen I↓; collagen III↓; liver function↑	Antifibrotic factors (IGF‐1, IL‐6, HGF)↑	49
1.5 × 10^6^	Serum from rats with acute CCl_4_ injury	Pretreatment	MSCs	Intrahepatic	CCl_4_	Rats	Adipose	Fibrosis↓; liver functions↑	Hepatogenic differentiation↑	52
5 × 10^6^	BFGF	Pretreatment	MSCs	Caudal vein	CCl_4_	Rats	Adipose	Therapeutic effects on liver fibrosis↑	HGF↑	53
1 × 10^4^/cm^2^	Diode laser and HGF	Pretreatment	MSCs	Tail vein	CCl_4_	Mice	Umbilical cord	Periportal fibrosis↓	Vascular congestion↓; mononuclear cellular infiltration↓; hepatocyte apoptosis↓	54
1.5 × 10^6^	Melatonin	Pretreatment	MSCs	Tail vein	CCl_4_	Rats	Bone marrow	Homing ability of MSCs↑; liver function↑	The interaction of melatonin receptors and matrix enzymes	55
1 × 10^6^	Simvastatin	Cotreatment	MSCs and recipient preconditioning	Intrahepatic	TAA	Rats	Bone marrow	Hepatic collagen distribution↓; hydroxyproline content↓; liver function↑	TGF‐β/Smad signalling↓; α‐SMA↓	56
2 × 10^6^	Icariin	Pretreatment	MSCs	Tail vein	CCl_4_	Mice	Umbilical cord	Progression into hepatic fibrosis↓	Antioxidant activities of MSCs↑	57
5 × 10^6^	Baicalin	Pretreatment and cotreatment	MSCs and recipient preconditioning	Subcutaneous	CCl_4_	Rats	Bone marrow	Fibrotic area↓; recovery of liver function↑	Liver inflammation↓	58
5 × 10^6^	Splenectomy	Pretreatment	Recipient preconditioning	Caudal vein	CCl_4_	Rats	Adipose	Liver function↑; Fibrotic progression↓	Migration rate of MSCs↑; SDF‐1α↑; HGF↑	60
5 × 10^6^	Hepatic irradiation	Pretreatment	Recipient preconditioning	Portal vein	TAA	Rats	Bone marrow	Liver function↑	Homing and repopulation of MSCs↑	61
1 × 10^6^	Sodium nitroprusside	Pretreatment	Recipient preconditioning	Intrahepatic	CCl_4_	Mice	Bone marrow	Fibrotic markers↓; cytokeratin 18↑; albumin↑; eNOS↑; liver fibrosis↓	MSCs homing↑	62
1 × 10^6^	IL‐6	Pretreatment	Recipient preconditioning	Intrahepatic	CCl_4_	Mice	Bone marrow	Antifibrotic effects↑; lactate dehydrogenase↓; apoptosis of hepatocytes↓	Bcl‐xl↑; Bax↓; caspase‐3↓; NFκB↓; TNF‐α↓	63
1.5 × 10^6^	Praziquantel	Pretreatment	Recipient preconditioning	Intrahepatic	*Schistosoma mansoni*	Mice	Wharton’s jelly	Liver fibrosis↓	α‐SMA↓; COL1A1↓; IL‐13↓	64
1 × 10^6^	BMSCs were preconditioned with SDF‐1α; rats were preconditioned with resveratrol	Pretreatment	MSCs and recipient preconditioning	Caudal vein	Common bile duct ligation	Rats	Bone marrow	Homing rate of MSCs into liver↑; homing rate of MSCs into lung and spleen↓; SIRT1↑; p53↓	Expression of CXCR4 and MMP‐9 in MSCs↑; Expression of AKTs and CXCL12 in injured liver↑	65

eNOS, endothelial nitric oxide synthase

## 7 PRETREATMENT OR COTREATMENT OF MSCS

Although hypoxia has been proved to activate STAT3/hypoxia‐inducible factor‐1α (HIF‐1α)/VEGF and stromal cell‐derived factor‐1 (SDF‐1α)/chemokine growth factor receptor (CXCR)‐4 signalling pathways and suggested to augment the recruitment of MSCs,^51^ the hypoxia preconditioned MSCs has never been transplanted into animal models with liver fibrosis. It may raise a hot topic to investigate the transplantation efficacy of hypoxic MSCs in vivo in times to come. 3D culture improved the release of antifibrotic factors including insulin growth factor 1 (IGF‐1), IL‐6 and HGF of MSCs, and 3D‐cultured MSCs significantly decreased the levels of collagen I and collagen III and improved liver function thus ameliorating hepatic fibrosis more effectively than general cultured MSCs.^49^ Pretreatment with serum from rats with acute CCl_4_ injury‐induced MSCs was done to express more hepatic markers including AFP, albumin, cytokeratin 8 and cytokeratin 19, and the pretreated MSCs markedly repaired the fibrosis and liver functions after 1 month of transplantation.^52^ Furthermore, pretreatment with basic fibroblast growth factor (BFGF) significantly improved the proliferation and differentiation of MSCs in vitro and markedly enhanced the therapeutic effects on liver fibrosis in vivo by enhancing the secretion of HGF in MSCs.^53^ Pretreatment with diode laser and HGF on MSCs improved the body and liver weights, reduced vascular congestion, mononuclear cellular infiltration, reduced hepatocyte apoptosis and minimized periportal fibrosis in CCl_4_‐induced liver fibrosis.^54^

Some special medicines which may serve as antioxidative agents and exert anti‐inflammatory function in vitro and in vivo, can significantly improve the therapeutic effects of MSCs in eliminating liver fibrosis. Melatonin, which serves as a regulator of circadian rhythms, highly improves the homing ability of MSCs and serves as a promising agent for improving the recovery of liver function in liver fibrosis models.^55^ The combination treatment of simvastatin plus MSCs decreased hepatic collagen distribution, lowered the hydroxyproline content and rescued liver function impairment by suppressing TGF‐β/Smad signalling and α‐SMA in HSCs.^56^ Pretreatment with icariin increases the antioxidant activities of injected MSCs and halts progression into hepatic fibrosis by accelerating the recovery of liver function.^57^ Baicalin, isolated from the root of *Scutellaria baicalensis* Georgi, was able to promote hepatogenic differentiation of MSCs into hepatocytes in vitro, and transplantation of baicalin‐treated MSCs and baicalin demonstrated good effects on reducing the fibrotic area and recovery of liver function and suppression of liver inflammation than MSC transplantation alone.^58^ Considering attaining consistent robust engraftment in the normal liver is an obstacle for MSC transplantation, magnetic targeting combined with high rate of proliferation in situ was demonstrated to significantly increase the initial dwell time of endoderm progenitor cells and increase the engraftment of them in the undamaged liver.^59^ Although magnetic targeting is a new method with little adverse effect on cell viability, whether this innovative method will enhance the engraftment of MSCs in animals with liver fibrosis should be further verified.

Preconditioning by different factors and medicine can influence the biological activity of MSCs ex vivo and in vivo, thereby improving their reparative efficacy for applications in current regenerative medicine.

## 8 PRECONDITIONING OF RECIPIENTS

In recent years, multiple strategies focusing on the preconditioning of recipients have been developed to improve the efficiency of MSCs in repairing liver injury and reversing liver fibrosis. Splenectomy prior to MSC transplantation can improve liver function and suppress fibrotic progression more efficiently than MSC transplantation alone by improving the migration rate of MSCs and up‐regulating plasma SDF‐1α and serum HGF in liver cirrhosis patients.^60^ Liver fibrotic rats received a hepatic irradiation preconditioning before MSC transplantation, and this strategy significantly enhanced the homing and repopulation of MSCs and improved liver function than the control group, for eliminating liver fibrosis.^61^ Pretreatment with sodium nitroprusside on CCl_4_‐injured mice improved the MSC homing rate, thus decreasing the expression of fibrotic markers including α‐SMA, collagen 1α1, TIMP, nuclear factor κB (NFκB) and inducible nitric oxide synthase (iNOS) and liver fibrosis.^62^ Pretreatment of fibrotic liver with IL‐6 significantly improves the survival rate of MSCs, thus providing more effective antifibrotic effects by improving glycogen storage,^63^ moreover, the pretreatment also demonstrated a significant reduction in lactate dehydrogenase release and apoptosis in hepatocytes via up‐regulation of Bcl‐xl and down‐regulation of Bax, caspase‐3, NFκB and TNF‐α.^63^ In *Schistosoma mansoni*‐induced liver fibrosis, oral praziquantel treatment further enhanced the beneficial effects of MSC transplantation on regression of liver fibrosis as demonstrated by down‐regulation of α ‐SMA, COL1A1 and IL‐13 in liver tissue and other parameters such as morphometric, histopathological and gelatin zymographic results.^64^

Another combined therapy significantly increased the homing rate of MSCs into the liver and decreased the homing rate of MSCs into the lung and spleen for elimination of liver fibrosis. Hajinejad et al pretreated MSCs in vitro with SDF‐1α to up‐regulate the secretion of CXCR‐4 and MMP‐9 and pretreated recipients with resveratrol to increase the levels of AKTs and CXCL12 in injured liver. In addition, the combined therapy also increased the expression of sirtuin (SIRT)‐1, but decreased the expression of p53 in the liver.^65^ Finally, some pretreatments or cotreatments may be tested in recipients to improve the therapeutic effects of MSC transplantation for the repair of liver injury in liver fibrosis.

## 9 GENE MODIFICATION

Gene modulation is generally applied to reprogram somatic cells into a stemness state, and it can effectively enhance the effects of MSC transplantation in liver fibrotic models as well (Table 2).

**Table 2:** Gene modulation effectively enhances the effects of MSC transplantation in liver fibrotic models

Pathogen	Animal	MSC source	MSC dose	Gene modification	Route	Effect	Potential mechanism	Ref
CCl_4_	Rats	Bone marrow	3 × 10^6^	↓TIMP‐1	Intravenous	Fibrotic area↓	TIMP‐1↓	66
CCl_4_	Rats	Bone marrow	3 × 10^6^	↑MMP1	Tail vein	Biochemical parameters↑; progression of liver fibrosis↓	MMP1↑	67
TAA	Mice	Bone marrow	5 × 10^5^	↑IGF‐I	Tail	Inflammatory responses↓; collagen deposition↓	IGF‐I↑; HGF↑	68
CCl_4_	Rats	Bone marrow	1 × 10^6^	↑HGF	Tail vein	Fibrosis area↑	Migratory ability of MSCs↑; responses to SDF‐1α↑	69
Dimethylnitrosamine	Rats	Bone marrow	1 × 10^7^	↑HGF	Spleen	Therapeutic effects of MSCs↑	TIMP‐1↓; PDGF‐bb↓;TGF‐β1↓; MMP‐9↑; MMP‐13↑; MMP‐14↑; urokinase‐type plasminogen activator↑	70
TAA	Mice	Adipose	1.5 × 10^6^	↑FGF21	Tail vein	Hyaluronic acid↓; fibrotic factors↓	p‐JNK↓; NF‐κB↓; p‐Smad2/3↓	71
CCl_4_	Mice	Umbilical cord	N/A	↓TGFβ‐1	Tail vein	Aminotransferases↓; fibrosis area↓	TGFβ‐1/Smad pathway↓	72
TAA	Rats	Bone marrow	1 × 10^6^	↑Decorin	Intrahepatic	Liver fibrosis↓	Proliferation of HSCs↓; TGF‐β/Smad signalling↓	73
CCl_4_	Rats	Bone marrow	2 × 10^6^	↑Urokinase plasminogen activator	Tail vein	Liver tissue fibrosis↓	Wnt signalling↓	74
CCl_4_	Mice	Bone marrow	N/A	↓Androgen receptor	N/A	Self‐renewal and migration abilities of MSCs↑	Activation of infiltrating macrophages and HSCs↓	75
CCl_4_	Mice	Adipose	1 × 10^5^	↑MiroRNA‐122	Tail vein	Collagen deposition↓; therapeutic effects of MSCs↑	Activation of HSCs↓	76

Considering the important role of TIMP‐1 in liver fibrosis progression, lentiviral vector‐mediated silencing of TIMP‐1 in MSCs significantly reduced fibrotic area and collagen deposition in a rat model of liver fibrosis.^66^ In addition, MSCs transfected with MMP‐1 enhanced the reduction of liver fibrosis than MSC group by down‐regulating collagen content and inhibiting activation of HSCs.^67^ Overexpression of IGF‐I in MSCs significantly increased the levels of IGF‐I and HGF in the livers of treated mice than MSC group, and multiple doses of modified MSCs dramatically suppressed inflammatory responses and reduced collagen deposition in fibrotic livers.^68^ Overexpression of HGF in MSCs also enhances the migratory ability of MSCs, and these modified MSCs have stronger responses to SDF‐1α than control MSCs.^69^ After transplantation, these modified MSCs further decreased the levels of hepatic TIMP‐1 level and the hepatic release of fibrogenic cytokines such as platelet‐derived growth factor (PDGF)‐bb and TGF‐β1 while increasing the hepatic levels of MMP‐9, MMP‐13, MMP‐14 and urokinase‐type plasminogen activator.^70^ FGF21‐secreting MSCs can produce more α‐lactalbumin and lactotransferrin and inhibited TGF‐β1‐induced expression of α‐SMA and collagen in LX‐2 cells. They significantly eliminated liver fibrosis by down‐regulating hyaluronic acid and reducing the release of fibrotic factors such as α‐SMA, collagen and TIMP‐1 by inhibition of p‐JNK, NF‐κB and p‐Smad2/3 signalling.^71^ TGFβ‐1‐siRNA significantly improved the repair potential of MSCs against hepatic injury through TGF‐β1/Smad pathway, accompanied by down‐regulation of aminotransferases and reduced fibrosis area.^72^

Decorin plays a protective role against fibrogenesis by modulating the degradation of the extracellular matrix. MSCs infected with decorin‐expressing adenovirus more effectively impeded the development of thioacetamide (TAA)‐induced liver fibrosis in rat models than unmodified MSCs by inhibition of the TGF‐β/Smad signalling pathway.^73^ MSC administration significantly attenuates, and MSCs transfected with adenovirus‐mediated human urokinase plasminogen activator further reduced the extent of liver tissue fibrosis via down‐regulation of the Wnt signalling pathway.^74^ Knockout of androgen receptor in MSCs results in enhancement of self‐renewal and migration abilities of MSCs and consequently suppressed the infiltrating macrophages and HSC activation.^75^ MicroRNAs or non‐coding RNAs target mRNA for degradation or inhibition and may determine the migration and therapeutic effects of MSCs. Although lentivirus‐mediated overexpression of miroRNA‐122 does not alter the phenotype or differentiation potential of adipose‐derived MSCs in vitro, overexpression of microRNA‐122 effectively suppresses the activation of HSCs and eliminates collagen deposition in liver tissue, thus improving the therapeutic effects of MSCs.^76^

Gene modification influences MSC activities including differentiation, paracrine pathway, proliferation, survival and migration, thereby consequently influences MSC transplantation efficacy. Although genetically modified MSCs significantly reduce fibrogenesis and repair liver dysfunction, the low efficacy of genetic modification and its potential for tumourigenicity may limit its application.

## 10 CLINICAL TRIALS

Investigators focus on developing new method to improve MSC efficiency in vivo in animal models with liver cirrhosis, but the real effects should be estimated by clinical trials (Table 3). The Model for End‐stage Liver Disease (MELD) score is widely used in experimental studies and clinical applications to evaluate the degree of severity of the chronic liver disease; indicators including serum creatinine, total bilirubin and international normalized ratio are commonly used to estimate the prognosis of patients with chronic liver diseases.^77^

**Table 3:** Clinical trials of MSC transplantation in patients with liver fibrosis

Total sample size	Test group	Control group	Type	Treatment	Control	Dose	Effect	Follow‐up	Ref
39	20	19	HBV‐induced liver fibrosis	MSCs+entecavir	Entecavir	8.45 ± 3.28 × 10^8 ^cells/patient	Treg cells↑; Foxp3↑; TGF‐β; Th17 cells↓; RORγt↓; IL‐17↓; TNF‐α↓; IL‐6↓	24 wk	1
6	6	0	HCV‐induced liver fibrosis	MSCs	N/A	1 × 10^6^ cells/kg	Jaundice symptoms↓; ALT↓; AST↓; bilirubin↓	6 mo	2
27	15	12	Decompensated cirrhosis induced by HBV, HCV, PBC, autoimmune hepatitis and other reasons	MSCs	Placebo	A median of 1.95 × 10^8^ cells/patient	No significant improvement	12 mo	78
45	30	15	HBV‐induced decompensated liver cirrhosis	MSCs	Saline	0.5 × 10^6 ^cells/kg	Albumin↑; total serum bilirubin↓; end‐stage liver disease scores↓; ascites↓	1 y	79
103	50	53	HBV‐induced decompensated liver cirrhosis	MSCs+normal medical treatment	normal medical treatments	(4.0‐4.5) × 10^8^ cells/patient	IL‐6↓; TNF‐ α ↓; T8 cells↓; B cells↓; IL‐10↑; T4 cells↑; Treg cells↑; Child‐Pugh scores ↓; mortality rate↓; no remarkable differences in the incidence of developing liver failure	36 wk	80
25	15	10	HCV‐induced liver cirrhosis	MSCs/hepatogenic MSCs	Supportive treatment	1 × 10^6^ cells/kg	Prothrombin↑; albumin↑; bilirubin↓; MELD score↓	6 mo	81
12	12	0	Alcoholic cirrhosis	MSCs	N/A	5 × 10^7^ cells/patient	Histological analysis↑; Child‐Pugh score↓; TGF‐β1↓; COL1A1↓; α ‐SMA↓	12 wk	82
72	48	24	Alcoholic cirrhosis patients who had been abstaining from alcohol for more than 6 mo	MSCs	Supportive treatment	5 × 10^7^ cells/patient	Collagen deposition↓; fibrosis quantification↓	12 mo	83
7	7	0	PBC patients with a suboptimal response to UDCA treatment	MSCs	N/A	0.5 × 10^6^ cells/kg	Alkaline phosphatase↓; γ‐glutamyltransferase↓; fatigue and pruritus↓	48 wk	84
10	10	0	PBC patients	MSCs+UDCA	N/A	(3‐5) × 10^5 ^cells/kg	ALT↓; AST↓; γ‐glutamyltransferase↓; immunoglobulin M↓; CD8^+^ T cells↓; CD4^+^CD25^+^Foxp3^+^ T cells↑; IL‐10↑; quality of life↑	12 mo	85
26	26	0	Autoimmune disease‐induced liver cirrhosis	MSCs (umbilical cord MSCs; cord blood‐derived MSCs; bone marrow‐derived MSCs)	N/A	1 × 10^6^ cells/kg	Albumin↑; prothrombin time↓; MELD score↑	2 y	86
60	30	30	Hepatolenticular degeneration‐induced liver fibrosis	MSC transplantation+penicillamine	Penicillamine	6 × 10^6 ^cells/patient	HA↓; PCIII↓; LN↓; CIV↓; TIMP‐1↓; MMP‐1↓	12 wk	87
50	50	0	Alcoholic cirrhosis, HBV‐induced liver fibrosis, HCV‐induced liver fibrosis	MSCs	N/A	3 × 10^7^ cells/patient	Albumin↑; prealbumin ↑; MELD scores ↓; no improvement in coagulation indicators or AFP	24 wk	88

Although a randomized controlled trial with several patients demonstrated that autologous MSC transplantation through peripheral veins exerted no beneficial effects on the liver function of cirrhotic patients,^78^ there are still various studies which proved that MSC transplantation benefits liver cirrhosis patients. In one such study involving 56 patients with HBV‐related liver fibrosis, MSC transplantation after basal entecavir treatment was more effective than control treatment, thus significantly increasing the number of Treg cells and the level of a Treg‐related transcription factor (Foxp3), while decreasing the number of Th17 cells and the level of Th17‐related transcription factor (RORγt). Furthermore, MSC implantation significantly improved serum TGF‐β levels but decreased the serum levels of IL‐17, TNF‐α and IL‐6 after transplantation in vivo in HBV‐related liver cirrhosis patients.^1^ Zhang et al enrolled patients with HBV‐induced decompensated liver cirrhosis, treated them with MSC transplantation and followed up to assess the long‐term efficacy. After one year, MSC transfusion proved to be clinically safe and effectively reduced ascites in these patients.^79^ Conversely, Fang et al enrolled 103 HBV‐induced decompensated liver cirrhosis recently and showed that MSC transplantation significantly decreased the serum levels of IL‐6, TNF‐α, T8 cells and B cells, but increased the serum levels of IL‐10, T4 cells and Treg cells at 2 and 4 weeks after transplantation. Although MSC transplantation improved the Child‐Pugh scores from 4 to 36 weeks after treatment and decreased the mortality rate during the follow‐up period, they demonstrated no remarkable differences in the incidence of developing liver failure in MSC group and control group.^80^ On the other hand, it has been shown that administration of MSCs reduces jaundice symptoms and decreases serum levels of aminotransferases and bilirubin in patients with HCV‐induced liver fibrosis.^2^ In addition, 25 patients with Child C grade HCV‐induced liver cirrhosis underwent MSC transplantation and hepatogenic MSC transplantation (approximately 40% hepatocyte‐like cells and 60% MSCs), both treatments up‐regulated the levels of prothrombin and albumin while down‐regulating the levels of bilirubin and MELD scores comparably.^81^

In addition to the most common types of liver cirrhosis, MSC transplantation has also been clinically applied to alcoholic cirrhosis, autoimmune liver cirrhosis and hepatolenticular degeneration. MSC transplantation dramatically improved the histological analysis of patients with alcoholic cirrhosis and their Child‐Pugh scores, with a concurrent decrease in the levels of TGF‐β1, COL1A1 and α‐SMA.^82^ In addition, 72 patients with alcoholic cirrhosis were enrolled for MSC transplantation, and although one‐time and two‐time transplantation reduced collagen deposition by 25% and 37%, respectively, there was no significant difference in fibrosis quantification between the two groups.^83^ MSC transplantation has been demonstrated to neither influence the expression levels of total bilirubin, aminotransferases, albumin or immunoglobulin M nor affect international normalized ratio, prothrombin time activity or Mayo risk scores in PBC patients. It significantly alleviated the levels of serum alkaline phosphatase and γ‐glutamyltransferase and relieved symptoms including fatigue and pruritus in the same population.^84^ Wang et al argued that MSC transplantation down‐regulated the levels of ALT, aspartate aminotransferase (AST), γ‐glutamyltransferase, immunoglobulin M and CD8^+^ T cells and up‐regulated the levels of CD4^+^CD25^+^Foxp3^+^ T cells and IL‐10 in PBC patients; moreover, the quality of life of these patients were improved significantly, as demonstrated by PBC‐40 questionnaires.^85^ Liang et al enrolled 26 patients with autoimmune disease‐induced liver cirrhosis and observed beneficial effects on liver function after infusions of MSCs via peripheral veins with no serious adverse effects.^86^ Zhang enrolled 60 patients with hepatolenticular degeneration‐induced liver fibrosis and demonstrated that MSC transplantation significantly improved liver function and enhanced the therapeutic effect of penicillamine.^87^

In consideration to liver cirrhosis induced by various factors, MSC transplantation significantly increased the levels of serum albumin and prealbumin and improved MELD scores but exerted no significant influence on the levels of coagulation indicators or AFP in patients with decompensated cirrhosis (alcoholic liver disease, 37 with HBV infection and 2 with HCV infection).^88^ Sang et al displayed a meta‐analysis include a total of 14 trials and showed that the combination of MSCs and traditional supportive therapy not only improved the liver function of patients with liver cirrhosis induced by various factors but also improved their quality of life and clinical symptoms including fatigue, appetite, ascites and abdominal distension without severe adverse events.^89^ Although multiple studies have demonstrated efficacy in repairing liver function and improving the prognosis of liver fibrosis, it is worth noting that further studies must enrol more patients with liver fibrosis induced by different factors to better elucidate the safety and effects of MSC transplantation.

## 11 CONCLUSIONS

**Figure 2:**
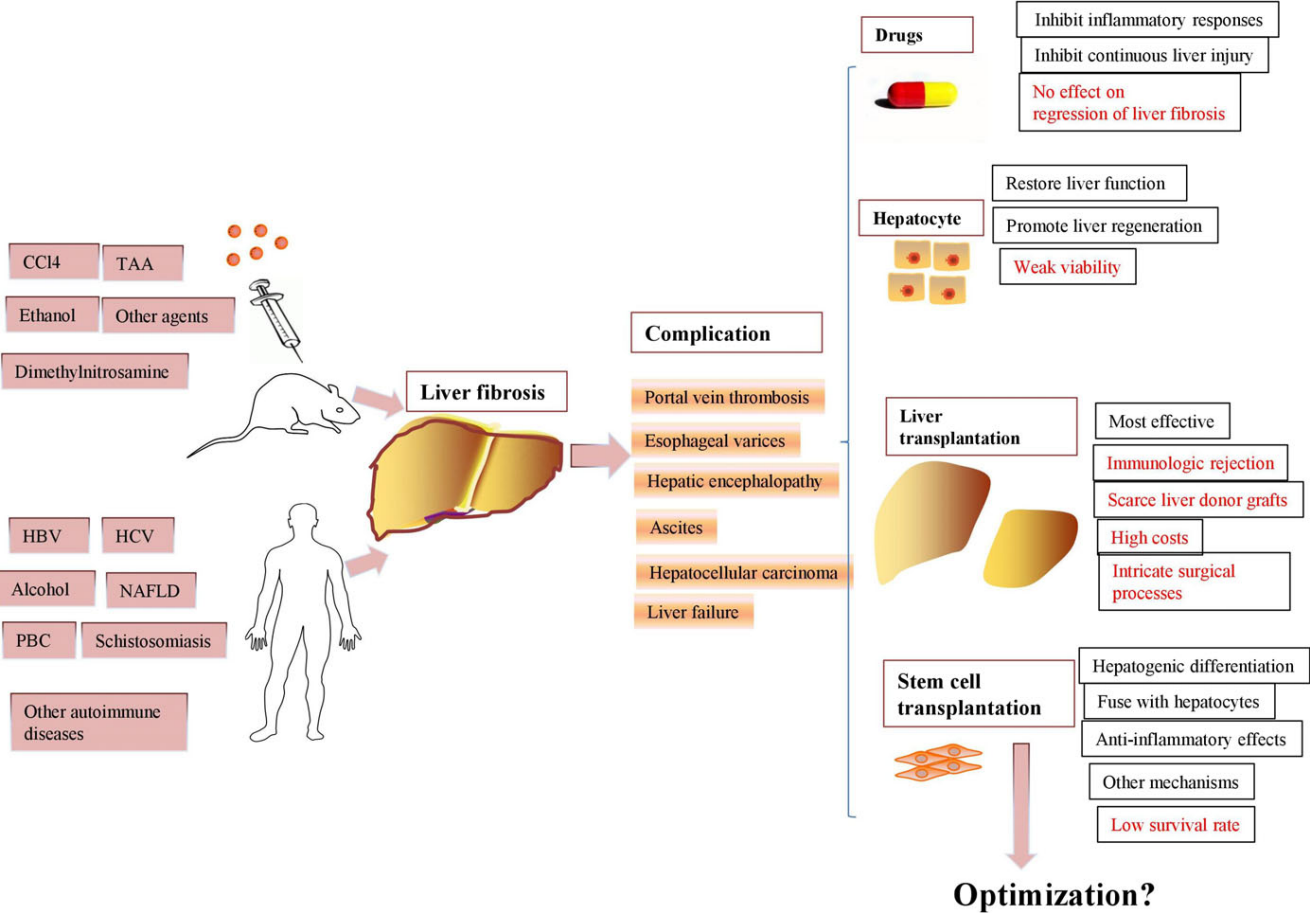
Drugs, liver transplantation, hepatocyte transplantation and stem cell transplantation can be applied to liver fibrosis induced by multiple toxic factors

Long‐term exposure to viral hepatitis, toxic chemicals, alcohol, lipid deposition, parasites or autoimmune elements in human and animals lead to liver fibrosis with a poor prognosis. The imbalance between MMPs and TIMPs is a key contributor to the pathogenesis of liver fibrosis, and HSC activation also plays a vital role in its progression. Drugs, liver transplantation, hepatocyte transplantation and stem cell transplantation can be applied to liver fibrosis induced by multiple toxic factors, while they have advantages and disadvantages, respectively (Figure 2). MSCs from different sources have multiple advantages including self‐renewal, anti‐inflammatory abilities, multipotency and antitumour abilities, thus further comparison for MSCs from various sources may help to find the optimal source for regression of liver fibrosis. MSC transplantation via the portal vein route currently seems to be the best choice for repairing liver function in liver disease. More and more studies focus on investigating the anti‐inflammatory effects and immunomodulatory effects of MSC‐derived sources including MSC‐based cell‐free therapy and hepatogenic MSC transplantation for eliminating the fibrotic content of liver tissue. Most of the all, after blind, randomized clinical studies are further established for larger populations with longer‐term follow‐up exams, we may draw the conclusion of the MSC transplantation effects on patients with liver fibrosis. Only by addressing these concerns will we anticipate to improve the therapeutic effects of MSC transplantation for liver regeneration to enhance the quality of life and prolong the survival time of patients with liver fibrosis.

## CONFLICT OF INTEREST

The authors declare no competing financial interests.
